# Age-Related and Gender-Related Increases in Colorectal Cancer Mortality Rates in Brazil Between 1979 and 2015: Projections for Continuing Rises in Disease

**DOI:** 10.1007/s12029-020-00399-8

**Published:** 2020-04-04

**Authors:** Francis L. Martin, Camilo L. M. Morais, Juliana Yumi Sakita, Sergio Akira Uyemura, Vinicius Kannen

**Affiliations:** 1 School of Pharmacy and Biomedical Sciences, University of Central Lancashire, Preston, UK; 2 Department of Genetics, University of Sao Paulo, Ribeirao Preto, Brazil; 3 Department of Toxicology, Bromatology, and Clinical Analysis, University of Sao Paulo, Ribeirao Preto, Brazil; 4 Lunenfeld-Tanenbaum Research Institute, Mount Sinai Hospital, Toronto, ON Canada

**Keywords:** Ageing, Cancer mortality, Epidemiology, Lifestyle

## Abstract

**Purpose:**

Brazil is the largest country in South America. Although a developing nation, birth rates have been decreasing in the last few decades, while its overall population is undergoing lifestyle changes and ageing significantly. Moreover, Brazil has had increasingly high mortality rates related to colorectal cancer (CRC). Herein, we investigated whether the Brazilian population is exhibiting increasing mortality rates related to colon cancer (CC) or rectal cancer (RC) in recent years.

**Methods:**

We examined data from the Brazilian Federal Government from 1979 to 2015 to determine whether CRC mortality and the population ageing process may be associated.

**Results:**

Our mathematical modelling suggests that mortality rates related to CC and RC events in the Brazilian population may increase by 79% and 66% in the next 24 years, respectively. This finding led us to explore the mortality rates for both diseases in the country, and we observed that the highest levels were in the south and southeast regions from the year 2000 onwards. CC events appear to decrease life expectancy among people during their second decade of life in recent years, whereas RC events induced decreases in life expectancy in those aged >30 years. Additionally, both CC and RC events seem to promote significant mortality rates in the male population aged > 60 years and living in the southern states.

**Conclusion:**

Our dataset suggests that both CC and RC events may lead to a significantly increasing number of deaths in the Brazilian male population in coming years.

## Introduction

Colorectal cancer (CRC) is currently reported as one of the leading causes of cancer-related deaths worldwide, in males and females [1]. One should consider that CRC terminology comprises two types of cancer with similar risk factors: colon cancer (CC) and rectal cancer (RC). The colon has two anatomical components referred to as the proximal and distal, which respectively connects this organ to the small intestine and the rectum. The rectum connects the distal colon to the anal sphincter [2]. Both CC and RC have significant genetic similarities regarding the mutations promoting their development. Symptoms are quite similar for both diseases, although bleeding in CC patients may be observed in brown or black colours, while RC events are related to brighter red bleedings. Because of anatomical patterns, RC is prone to metastasize to the thorax, bones, and nervous system, while the peritoneum seems to be a recurrent target for CC metastases. Also, cancer recurrence seems to be more frequent in RC patients. Moreover, RC events seem to be somewhat more common in men than women [3]. We should stress that because aetiology and risk factors are almost the same for both CC and RC events, they are generally widely known together as CRC [1, 2, 4, 5].

Until the first half part of the last century, CRC was a rather rare disease even in countries currently with high rates [4]. Considering that most CRC cases are sporadic in aetiology, changes in lifestyle across the globe could thus be directly linked to the increasing incidence of this disease [1, 4, 6–8]. Although an uncountable number of factors may augment CRC rates in a given human cohort, ageing of the worldwide population may play the most pivotal role in this disease aetiology and can be considered one of the primary mechanisms driving the CRC development [5]. For instance, most CRC incidence and mortality occurs in elderly populations living in developed countries [6].

In keeping with this idea, Kuipers et al. observed that the average lifetime risk for CRC might be up to 5% in a general population set without a family history for the disease. In cases where a first-degree family member >50 years old has been diagnosed with CRC, this lifetime risk may double, while it could even triple if the first-degree relative were <50 years of age by the time of diagnosis [4]. However, on analysing CRC rates in Americans aged <50 years old, an increasing incidence of this disease in the younger US adult population is revealed [7, 9]. Interestingly, it has been reported that CRC incidence declined in the USA, generally [1]. On the contrary, another study suggests that about 60% of CRC cases will be found in developing countries by the year 2030 [8].

Brazil is the largest country in South America and has the fifth largest population worldwide. The elderly Brazilian population has been projected to achieve 64 million seniors by 2050 [10]. The country also faces significant social inequality among its elderly citizens, a factor that may impact on the incidence of chronic diseases and healthcare costs shortly [11]. The CRC incidence in Brazilians aged less than 50 years old has also been reported increased by 35% in recent years [12]. Although developed countries have shown a significant decrease in CRC death rates [1], the Brazilian population endured increased numbers of CRC-related deaths for the last 30 years [13]. In as much as CRC mortality rates vary in between different Brazilian states, a factor closely related to the economic development of each region, CRC mortality rates have significantly increased over recent years in the country [14]. These facts raise some concern that the Brazilian population could have a significant increase in CRC-related rates for both incidence and mortality soon, meaning that epidemiological studies on what regions have recently had the highest CRC mortality rates in the country are in significant need now. Herein, we studied the trend for CC and RC mortality during the next 24 years (from 2016 up to 2040), verified mortality rates for both diseases in different Brazilian regions throughout 36 years, as well as studied recent mortality rates according to age and gender.

## Material and Methods

### Data Collection

CRC mortality data were obtained from the Mortality Information System (SIM) of the Brazilian Ministry of Health, while population-based data were collected from the Brazilian Institute of Geography and Statistics (IBGE). According to the International Classification of Diseases (10^th^ revision, ICD-10) for CC (ICD-10, C18) and RC mortality rates (ICD-10, C19–21), mortality data were collected from 1979 to 2015 on an annual basis. According to the same timeframe, population-based data were also collected. This period has been chosen to further explore previous findings on CRC mortality rates in Brazil.

To statistically compare the Brazilian regions with the highest CRC mortality rates among themselves (Midwest [MW], Southeast [SE], and South regions [S]), we collected data that provides from regional health centres (RHCs). According to SIM, RHCs are government units responsible for coordinating the activities of the State Health Secretariat at the regional level of each Brazilian state. Thus, each Brazilian region is composed of different states with multiple RHCs in each one: Midwest region (Federal capital region [DF] had 1 RHC, Goias state [GO] had 16 RHCs, Mato Grosso do Sul [MS] had 12 RHCs, and Mato Grosso [MT] had 16 RHCs), Southeast region (Espirito Santo [ES] had 4 RHCs, Minas Gerais [MG] had 16 RHCs, Rio de Janeiro [RJ] had 9 RHCs, and Sao Paulo [SP] had RHCs), and South region (Parana [PR] had 16 RHCs, Santa Catarina [SC] had 16 RHCs, and Rio Grande do Sul [RS] had 16 RHCs).

### Statistical Analyses

Prospective values for mortality from 2016 until 2040 were calculated according to a prediction function *m*(*y*) described as follows:

1 $$ m(y)=f(y)\frac{dp(y)}{dy}{\left(\frac{d{p}_s(y)}{dy}\right)}^{-1}\frac{o_{65}(y)}{o_{65}(2015)} $$

where *f*(*y*) is a second-order polynomial function fitted to proportional mortality rates (PMR) recorded between the years *y* (1979 and 2015). This function represents the mortality growth regardless of factors such as population growth and ageing. Then, *p*(*y*) is a second-order polynomial function fitted to the official estimate of the Brazilian population between 2014 and 2016. This function represents how a population will grow over time. To this end, *p*_*s*_(*y*) is a linear function fitted to the official records of the Brazilian population between 2010 and 2015, which represents the population growth in the years anticipating the future estimation. We should also consider that *o*_65_(*y*) is the official percentage of people >65 years for the year *y*, while *o*_65_(2015) is the percentage of people >65 years in 2015 (the year that recorded data ends). The mortality function described in Eq. 1 was therefore adjusted to take into consideration variations in population growth and ageing over time. In summary, 1% of CC or RC mortality rates calculated by Eq. 1 means that 1% of the deaths in a determined year was caused by one of these diseases.

CC and RC mortality values were calculated as PMR, as described by Swaroop and Uemura [15]. One should note that any potential misclassification at diagnosis was not exposure-dependent, cause of death is precisely registered, all deaths are included, and population comparisons are consistent within the study [16]. According to Romeder and McWhinnie [17], years of potential life lost (YPLL) *per* 100,000 were determined by the number of deaths in the Brazilian population across different age ranges (between 15 and 79 years old) from 1979 to 2015. This calculation provides age-adjusted rates within a closed population. We further calculate the age-adjusted death rates (AADR) *per* 100,000 related to CC and RC events, according to the previous description of Yerushalmy (1951) [18]. These values were determined based on data from the regional health centres (RHCs) within each Brazilian state in the Midwest, Southeast, and South regions. Values have been adjusted to the Brazilian population of 2010.

Data analyses determined differences in mortality rates related to CC and RC events across different time points in various Brazilian regions by employing a two-way ANOVA model followed by the correction of multiple comparisons through the control of false discovery rates according to the two-stage step-up method of Benjamini, Krieger, and Yekutieli. These analyses were performed on MATLAB R2014b (MathWorks, Inc., Natick, MA, USA) and GraphPad Prism 8.1.0 (GraphPad, San Diego, CA, USA). *P* values of <0.05 were considered significant.

## Results

### CC and RC Mortality Across Different Brazilian Regions from 1979 to 2015

**Fig. 1:**
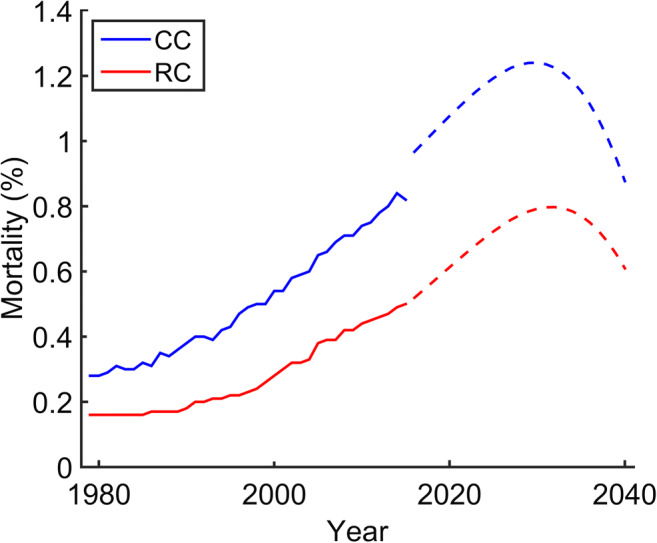
Mortality rates related to colon (CC) and rectal cancer (RC) in Brazil. While the recorded period (1979–2015) is shown as continuous lines for both diseases, dashed lines represent the data collected from perspective calculations between 2016 and 2040

First, we analysed the mortality rates related to CC and RC events in Brazil. We observed that the number of deaths by CC and RC increased 2.8-fold and 3.1-fold throughout the last 36 years, respectively. Indeed, our mathematical modelling projected an upward trend in mortality for both diseases in the forthcoming 24 years (CC, from 6.2 to 7.8 per 100,000; RC, from 3.6 to 5.4 per 100,000; Fig. 1; Table 1).

**Table 1:** Colon cancer (CC) and rectal cancer (RC) mortality rates *per* 100,000 in Brazil

Year	CC-related mortality	RC-related mortality
2010	4.71	2.80
2012	4.95	2.92
2014	5.35	3.12
2016	6.20	3.33
2018	6.61	3.66
2020	7.06	4.02
2022	7.52	4.39
2024	8.00	4.79
2026	8.46	5.18
2028	8.87	5.55
2030	9.19	5.87
2032	9.38	6.11
2034	9.39	6.22
2036	9.16	6.17
2038	8.64	5.92
2040	7.80	5.42

Values shown herein represent the recorded period between 2010 and 2014 for both CC and RC mortality rates, while a mathematical modelling projects these events from 2016 until 2040

**Fig. 2:**
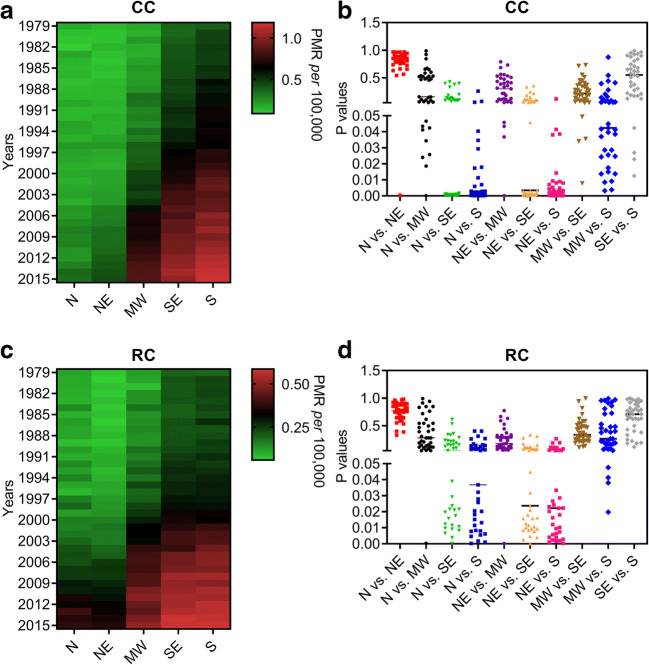
Mortality rates for colon cancer (CC) and rectal cancer (RC) in Brazil. **a** and **b** Heatmaps illustrate proportional mortality rates (PMR) for CC (**a**) and RC (**b**) according to a population of 100,000 people in different regions of Brazil (North [N]; Northeast [NE]; Midwest [MW]; Southeast [SE]; South [S]) from 1979 until 2015. Whether bright green colours represent the lowest mean values of proportional mortality rates (PMR) in each Brazilian region, bright red colours represent the highest values. **c** and **d** Scatter dot plots show *P* values that have been calculated by the two-way ANOVA model followed by correction of multiple comparisons through the control of false discovery rates according to the two-stage step-up method of Benjamini, Krieger, and Yekutieli

This prospective analysis led us to investigate mortality rates by CC and RC in different regions of the country. Our dataset shows that the population living in the MW, SE, and S regions of Brazil exhibit a significant increase in CC and RC mortality rates in recent years when compared with the inhabitants of the North (N) and Northeast (NE) regions (Fig. 2). We also observe that RC-related mortality in the MW population increased to a level no different from that of the SE region (Fig. 2c, d). Whether CC-related mortality is higher in the S region than in the SE, no difference has been found for death rates related to RC events between both areas (Fig. 2).

### Effects of Ageing on CC and RC Mortality in Recent Years

**Fig. 3:**
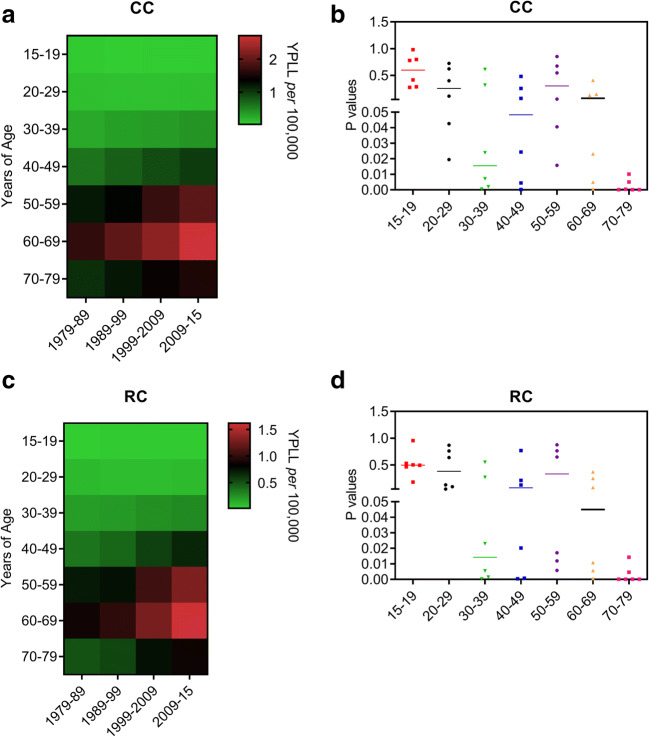
Years of potential life lost (YPLL) due to colon cancer (CC) and rectal cancer (RC) events in Brazil. **a and b** Heatmaps illustrate YPLL values for CC (**a**) and RC (**b**) are shown in different populations with age ranging from 15 to 79 years old between 1979 and 2015 in Brazil. Whether bright green colours represent the lowest mean values of YPLL, bright red colours represent the highest values. **c** and **d** Scatter dot plots show *P* values that have been calculated by the two-way ANOVA model followed by correction of multiple comparisons through the control of false discovery rates according to the two-stage step-up method of Benjamini, Krieger, and Yekutieli

To better understand these events, we calculated the impact on life expectancy due to CC and RC events in the Brazilian population. Both CC and RC elevated the most shortening in life expectancy between the years 2009 and 2015 (Fig. 3). We further found that the decrease in life expectancy associated with CC started during the second decade of life, while RC induced similar effects in subjects not younger than 30 years old (Fig. 3).

**Fig. 4:**
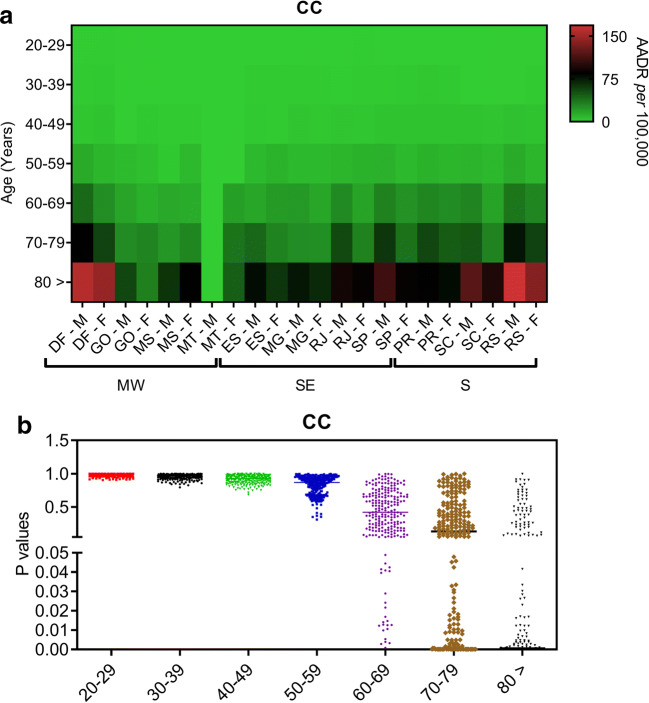
Age-adjusted death rates (AADR) related to colon cancer events (CC) during the year of 2015 are shown for both male (M) and female populations (F) living in different Brazilian states according to their regional location in the country. **a** AADR values related to CC are shown in different populations with age ranging from 20 to 80 years old during the year of 2015 in Brazil. These values were determined based on data from regional health centres (RHCs) within each Brazilian state in the Midwest (MW; Federal capital region [DF; RHC = 1], Goias state [GO; RHC = 16], and Mato Grosso do Sul [MS; RHC = 12] and Mato Grosso [MT; RHC = 16] states), Southeast (SE; Espirito Santo [ES; RHC = 4], Minas Gerais [MG; RHC = 16], Rio de Janeiro [RJ; RHC = 9], and Sao Paulo [SP; RHC = 16] states), and South regions (S; Parana [PR; RHC = 16], Santa Catarina [SC; RHC = 16], and Rio Grande do Sul [RS; RHC = 16]). Values have been adjusted to the Brazilian population of 2010. Whether bright green colours represent the lowest mean values of AADR in each Brazilian region, bright red colours represent the highest values. **b** Scatter dot plots show *P* values that have been calculated by the two-way ANOVA model followed by correction of multiple comparisons through the control of false discovery rates according to the two-stage step-up method of Benjamini, Krieger, and Yekutieli

**Fig. 5:**
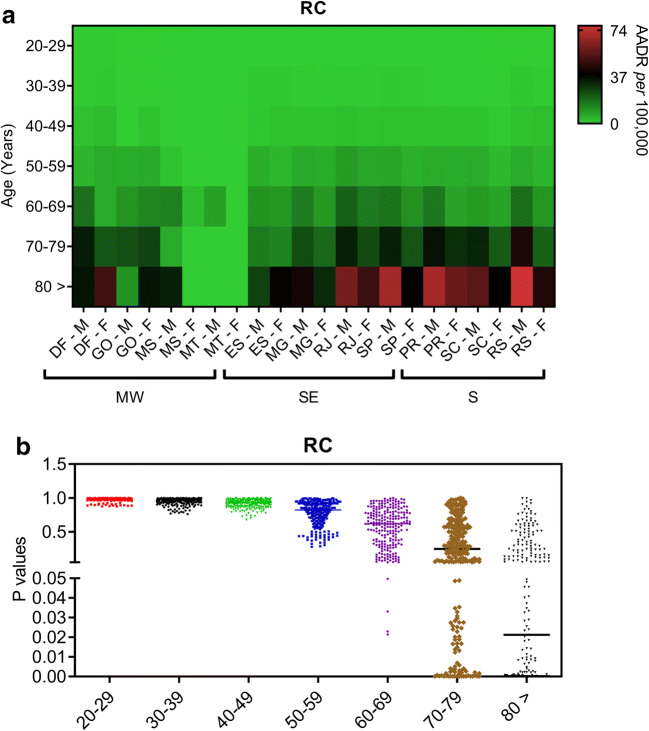
Age-adjusted death rates (AADR) related to rectal cancer events (RC) during the year of 2015 are shown for both male (M) and female populations (F) living in different Brazilian states according to their regional location in the country. **a** AADR values related to RC are shown in different populations with age ranging from 20 to 80 years old during the year of 2015 in Brazil. These values were determined based on data from regional health centres (RHCs) within each Brazilian state in the Midwest (MW; Federal capital region [DF; RHC = 1], Goias state [GO; RHC = 16], and Mato Grosso do Sul [MS; RHC = 12] and Mato Grosso [MT; RHC = 16] states), Southeast (SE; Espirito Santo [ES; RHC = 4], Minas Gerais [MG; RHC = 16], Rio de Janeiro [RJ; RHC = 9], and Sao Paulo [SP; RHC = 16] states), and South regions (S; Parana [PR; RHC = 16], Santa Catarina [SC; RHC = 16], and Rio Grande do Sul [RS; RHC = 16]). Values have been adjusted to the Brazilian population of 2010. Whether bright green colours represent the lowest mean values of AADR in each Brazilian region, bright red colours represent the highest values. **b** Scatter dot plots show *P* values that have been calculated by the two-way ANOVA model followed by correction of multiple comparisons through the control of false discovery rates according to the two-stage step-up method of Benjamini, Krieger, and Yekutieli

We then calculated mortality rates adjusted by age to clarify these concerning findings in recent years. The AADR for male and female inhabitants from the MW, SE, and S regions of Brazil between 2014 and 2015 reveal that southern men and women aged 70 years old or more exhibited higher mortality rates related to CC than those living in the SE and MW regions (Fig. 4). While southern men in their seventieth decade of life onwards had the highest RC mortality rates, this same effect appeared to occur in the female population aged ≥80 years old (Fig. 5). Indeed, the southern male population died more frequently of CC and RC than women following their seventieth decade of life (Figs. 4 and 5). Similar differences between male and female populations for CC and RC mortality rates are observed in the SE region (Figs. 4 and 5). Irrespective of whether the MW male population exhibited the highest CC mortality rates following their eightieth decade of life (Fig. 4), RC promoted the highest mortality among female inhabitants of this area (Fig. 5).

Studying the MW states, we found that the oldest male and female populations living in the Federal District (DF) had the highest CC and CRC mortality rates in the region (Figs. 4 and 5). Interestingly, those men aged 70 years old or more and residing in the state of Mato Grosso (MT) showed the lowest values of CC and RC mortality (Figs. 4 and 5).

In the SE region, men aged ≥80 years old and living in the states of São Paulo (SP) and Rio de Janeiro (RJ) showed the highest CC and RC mortality rates (Figs. 4 and 5). It seems that the old male population in the state of Espirito Santo (ES) had the lowest values of RC mortality (Fig. 4). In comparison with the states of São Paulo (SP) and Rio de Janeiro (RJ), women residing in the state of Minas Gerais (MG) had lower rates of CC and RC mortality (Figs. 4 and 5). Indeed, the male population living in the state of SP died more of CC and RC than women from 70 years old age onwards (Fig. 5).

Following the seventieth decade of life, both men and women living in the southern state of Rio Grande do Sul (RS) had the highest mortality rates of CC in the region (Fig. 4). Whether the Rio Grande do Sul (RS) male population had the highest mortality values due to RC events (Fig. 5), women living in the state of Parana (PR) showed the highest mortality for this disease when compared to the female population of other southern states (Fig. 5).

## Discussion

Cancer mortality rates for both CC and RC cases have significantly increased over the years in Brazil. One should note that CC mortality was higher than RC-related deaths. Since RC events have been widely associated with poor patient survival [3], we believe that the Brazilian population has a higher incidence of CC than RC-related events. Arnold and colleagues have analysed CC and RC mortality rates in 42 countries and reported similar findings to ours in Brazil [19].

Our findings also suggest that CC and RC mortality rates may rise even higher in the coming years. This finding is in keeping with what has been reported by previous studies for a time range similar to ours [8, 19, 20]. Moreover, Jemal and colleagues predicted the number of CRC-related deaths worldwide (881,000 events) and ranked this disease second among the malignancies most related to fatal cases in 2018 [1]. Indeed, increased incidence and mortality rates of this disease have been suggested as a marker of socioeconomic development [1]. Bray et al. suggest that Brazil is one of those nations where CRC incidence and mortality have increased in recent years [1].

In contrast, countries observed to have a decreasing incidence and mortality rates have been the USA, Japan, and France [21]. In the USA and Japan, this effect could partially be related to the implementation of long-standing screening and early detection programmes against CRC [22]. However, food-related issues promoting obesity in the younger American adult population has also been suggested to be associated with increasing CRC incidence in patients aged < 50 years old [7, 9].

Analysing CC and RC mortality rates over time and across different Brazilian regions suggests that potential changes in lifestyle have occurred in recent years. This notion seems more evident when analysing YPLL rates found in the Brazilian population. One should consider that changes in diet, body weight, and lifestyle could explain trends towards high CRC incidence, while best practices in cancer management and treatment may reduce mortality for this disease [21]. Besides the fact that a sound healthcare system decreases the number of cancer-related deaths, lifestyle profoundly impacts on both cancer risk and survival of patients. India seems to illustrate how changes in lifestyle impacts on CRC incidence and mortality. GLOBOCAN-based studies show that whether CRC incidence and mortality were lower in India than in Brazil by 2012 [21], analysing the same patterns 6 years later highlights a significant elevation of this disease in the Indian male population [1]. An Indian research group recently suggested that healthier lifestyle habits could reduce CRC incidence in the country [23]. In Brazil, research groups have been reporting frequent physical inactivity in the younger population, increasingly high intakes of red meat, and low consumption of fruits overall [24–27]. Folgueira and colleagues analysed 88 cases of gastric cancer and found that poor eating habits could be related to the development of this malignancy in younger adults living in Brazil [28].

The Brazilian population living in the southern regions of the country appear to exhibit the highest mortality rates for both CC and RC events. The male inhabitants of these Brazilian states may have a much higher mortality risk by either CC or RC than the female population. The Rio Grande do Sul (RS) state has previously been found to have the highest CRC mortality rates in Brazil [14]. Bray et al. have recently reported that the male population has a 50% higher chance of dying by any type of cancer than women worldwide [1]. A previous study carried out in the RS state revealed CRC to be the most prevalent type of cancer in both sexes combined, as prostate malignancies are the most common in men and breast cancer in women [29]. Although heredity impact on CRC risk even in Brazil [30], two independent studies have shown that only specific rare mutations may impact a small percentage of the Brazilian population in general [31–33]. Assunção and colleagues reported that processed meats are now consumed very regularly by the Rio Grande do Sul (RS) inhabitants [34]. The Brazilian population seems to have acquired unhealthy eating habits in recent years [24, 25, 27, 28]. Consumption of red or processed meat seems to be an established risk factor promoting CRC [35]. Since the implementation of longer-standing screening and early detection programmes, CRC mortality seems to have decreased in other countries [22]. Frasca and colleagues report that testing for faecal occult blood could provide a significant strategy for early detection of CRC in the Brazilian population [36].

We must further consider that the Brazilian population is rapidly ageing and that this process has the potential to increase the risk of cancer besides the threat imposed by an unhealthy lifestyle [5, 10]. Recent studies have demonstrated that ageing decreases proliferation rates but increases the mutational load in humans or any other given organism [37, 38]. Although these findings seem controversial at first sight, DeGregori suggested that ageing provides the required length of time required for oncogenic mutations to accumulate and cancer to arise in a particular body site [39]. While the Brazilian population is undergoing significant changes in lifestyle factors to closely mirror those in developed Western societies, expansion of its elderly population is coupled with a lack of efficient anticancer health programmes reported to lower CRC incidence in developed Western regions [1, 10, 11, 13, 20, 40]. Thus, it is no surprise that we report herein increasing CC and CRC mortality in this Brazil region currently and in the coming decades. The current Brazilian scenario unfortunately seems to be set ideally for exponential increases in CRC incidence and mortality in the coming decades.

Although our findings are in keeping with previous national and international forecasting that CRC incidence and mortality rates would increase in Brazil [8, 20], this current study has some limitations that should be considered. First, we must consider that the period required for data consolidation in a public database results in a few years gap from the time that death events have been registered until the release of data to the public. This secondary data usage further limits the study design, as well as the interpretation of these results at an individual level. Moreover, registration errors at SIM can, in a hypothetical scenario, lead to under- or overestimation of incidence and mortality rates of any given disease. Nevertheless, mortality trends can also be slightly modified in case new diagnostic and therapeutic guidelines are implemented in the country during the predicted time-lapse [13, 20, 40, 41].

Our current findings suggest that mortality rates for CC and RC are rapidly rising throughout many Brazilian regions. This scenario may worsen in the coming years, as Brazilians are readily acquiring dietary and lifestyle habits from Western developed countries that have a consistent pattern for high incidence and mortality related to CRC. The most needed attention regarding healthcare strategy in treatment and prevention should be given to the inhabitants of the RS state, as high rates of CC and RC mortality have been observed here for consecutive decades now. New studies on cancer-related deaths in the RS state are required now, as future findings may help to decrease mortality rates for other types of cancer in different regions of Brazil.
